# Comparison of metabolic rates of ropivacaine in cerebrospinal fluid as inferred from plasma concentrations between elderly patients and young patients

**DOI:** 10.1186/s13741-024-00372-0

**Published:** 2024-03-06

**Authors:** Dongshi Lu, Fei Cai, Yu Ming, Danqing Zhang, Demu Ba, Zhouyang Wu, Zhao Zhang

**Affiliations:** 1 Department of anesthesiology, Union Hospital, Tongji Medical College, Huazhong University of Science and Technology, 1277 Jiefang Avenue, Wuhan, 430022 People’s Republic of China; 2 College of Medicine and Health Science, Wuhan Polytechnic University, Wuhan, People’s Republic of China; 3 People’s Hospital of Bortala, Mongolian Autonomous Prefecture, Bole City, People’s Republic of China

**Keywords:** Ropivacaine, Cerebrospinal fluid (CSF), Hyaluronic acid (HA), Central lymphatic circulation (CLC), Postoperative cognitive dysfunction (POCD)

## Abstract

**Background:**

With the aging of human society, more and more elderly patients have to undergo surgery and anesthesia. Clinical observations have indicated from time to time that spinal anesthesia in the elderly appears to last longer than in young people, although there is limited research in this area and the mechanism is unclear at present time. This research work is expected to help understand the decline of local anesthetic metabolism in cerebrospinal fluid of elderly patients so as to help them with precise anesthesia and rapid rehabilitation.

**Methods:**

Twenty patients with spinal anesthesia in orthopedic lower limb surgery were selected to study the rate of drug metabolism in cerebrospinal fluid in two age groups, i.e.,18–30 years old and 75–90 years old. Ropivacaine in peripheral blood is used as a probe to reflect the speed of drug metabolism in cerebrospinal fluid. The contents of total Aβ protein and hyaluronic acid in the cerebrospinal fluid were investigated as well.

**Results:**

The equivalent dose of ropivacaine anesthetizes the elderly group for a longer time. The metabolism rate of ropivacaine in an elderly patient was slower than that of a young patient. No significant difference in total Aβ protein between the two groups was observed while hyaluronic acid in the elderly group was significantly higher than that in the young group.

**Conclusions:**

This study shows that the dose of ropivacaine should be reduced when used for anesthesia in elderly patients. The cumulation of ropivacaine and HA appears to imitate the degeneration of central lymphatic circulation metabolism in elderly people.

## Background

With the aging of human society, the number of operations for elderly patients is increasing year by year (Rump and Adamzik 2022). Clinical observations have indicated from time to time that spinal anesthesia in elderly people appears to last longer than in young people (Veering et al. 1987), yet there is limited research in this area with few relevant data available in the open literature and the mechanism still is unclear at present time. Total Aβ protein in CSF (cerebrospinal fluid) has been reported to be a substance that impairs cognitive function and its concentration could be a predictive indicator of cognitive impairment (Lanni et al. 2019). On the other hand, HA (hyaluronic acid) can be metabolized through lymphatic circulation and serves as a marker reflecting lymphatic circulation function (Jackson 2019). The meningeal lymphatic vessels have recently been recognized as an important player in cognitive impairment (Da Mesquita et al. 2018). The objective of this study is to investigate the impact of age on the metabolic rate of ropivacaine and the function of lymphatic circulation between elderly and young patients. The outcome of this research is expected to help understand the basis of a decrease in local anesthetic metabolism in the cerebrospinal fluid of elderly patients to guide anesthesiologists in providing precise anesthesia and rapid recovery for elderly patients.

## Materials and methods

### Patients

Before recruiting the patients, the protocol of this trial was approved by the Ethics Committee at Tongji Medical College (number: 2019-s1107).

From October 20, 2019, to October 30, 2019, twenty adult patients hospitalized at the Union Hospital of Tongji Medical College in Wuhan, China who were scheduled to undergo internal fixation of lower limb fracture were selected and assigned into two groups by age, a young group (18–30 years old, *n* = 10) and an elderly group (75–90 years old, *n* = 10), respectively, to receive spinal anesthesia. Exclusion criteria included: ASA III to V, past neurological and psychiatric disorders, consumption of tranquilizers, long-term use of psychotropic drugs, diabetes, renal disease, active liver disease, severe visual or auditory handicap, inability or unwillingness to abide by the protocol, loss of follow-up, and bleeding greater than 200 mL intraoperative.

### Anesthesia and surgery

Anesthesia method was practiced according to the routine of our hospital. Subarachnoid block was performed at the L_3–4_ vertebral interspace with 25 G spinal needle. A CSF sample of 2 mL was extracted from the subarachnoid space and stored at − 20 °C for future use. A ropivacaine (naropin, Astrazenca AB, SE -151 85, Sodertalje, Sweden) dose of 15 mg was then injected into the same subarachnoid space through the spinal needle without detaining a spinal catheter. The anesthesia level was adjusted to T_12_ level by adjusting the angle of head height and the fluctuation of blood pressure was maintained between 70 and 130% before operation. The heparin anticoagulant venous blood was extracted at 0.5, 1, and 2 h after spinal anesthesia. No sedative or analgesic drug was used during the operation. Anesthesiologists who do not know the research plan are responsible for recording the patient’s condition during the operation.

### Determination of total concentration of ropivacaine in plasma

The total concentration of ropivacaine was determined with high-performance liquid chromatography (Chen et al. 2020; Gromov et al. 2021) (HPLC, 2995 pump, 2996 diode array detector, Waters, USA). The HPLC column was a shim-pack C18 column (250 mm × 6 mm, 5 microns) and the pre-column was Alltech C18 (10 mm × 4.6 mm, 5 microns). The mobile phase was potassium dihydrogen phosphate/acetonitrile (75/25) buffer (pH 3.0). The column temperature was maintained at 25 ℃, flow rate at 1.0 mL/min, detection wavelength at 210 nm, and sample injection amount at 20 μL. Lidocaine (Zhaohui Pharmaceutical Co., Ltd., Shanghai, China) was used as the control substance in this work.

### Quantitative analysis of total Aβ protein and hyaluronic acid (HA) in CSF

Total Aβ protein concentration in CSF was determined using a bicinchoninic acid assay kit (Zhang et al. 2019) (P0010S, Beyotime Biotechnology, Nantong, Jiangsu Province, China). The optical densities were recorded using an enzyme mark instrument (SPECTRAMAX 190, Molecular Devices Corp., Sunnyvale, CA, USA) at 450 nm wavelength. A calibration curve was thus generated to calculate the protein concentrations in CSF.

The HA level in CSF was determined using an enzyme-linked immunosorbent assay kit for human HA according to the manufacturer’s instruction (Yoo et al. 2018) (ml064280, Shanghai Enzyme-linked Biotechnology Co., Ltd., Shanghai, China). The sample treatment was the same as with total Aβ protein. The optical density developed in proportion to the amount of specific cytokine was recorded using an enzyme mark instrument (SPECTRAMAX 190, Molecular Devices Corporation, Sunnyvale, CA, USA) at 450 nm wavelength. A calibration curve was then generated to calculate the concentration of HA in CSF.

### Statistical analysis

All data in this work were expressed as means ± S.D. Statistical analysis was conducted using SPSS 23.0 for Windows (SPSS Co., Ltd., Chicago, IL, USA). Baseline and demographic characteristics between the two groups were compared with paired *χ*^2^ or Yates’ corrected *χ*^2^ test for discrete variables and with independent samples *t* tests for continuous variables. A value of *P* less than 0.05 indicates that the difference is statistically significant.

## Results

### Patient population

Table 1 shows that there was no significant difference in height, weight, blood sugar, albumin level, liver, and kidney function between the two groups of patients studied in this work. The operation time, blood pressure, and heart rate were similar between the two groups. The maintenance time of anesthesia in elderly patients was significantly prolonged than that of the young ones. All patients have a good prognosis without delirium, though.

**Table 1:** Demographic characteristics of the patients studied

Group	Young group	Elderly group	*P* value
Age (year)	34 ± 5.76	75 ± 7.64*	< 0.001
Weight (kg)	65.15 ± 19.39	71.48 ± 22.56	0.420
Blood sugar (mmol/L)	5.3 ± 1.39	6.6 ± 1.74	0.283
Infusion volume (mL)	825 ± 125	795 ± 175	0.416
Albumin (g/L)	46 ± 6.63	43 ± 7.54	0.257
BUN (mmol/L)	5.48 ± 0.75	6.36 ± 1.21	0.247
CREA (μmol/L)	63.08 ± 16.33	68.66 ± 18.11	0.659
Blood pressure (MAP, mmHg)	94 ± 4.41	97 ± 11.1	0.674
Heart rate (bpm)	76.4 ± 11.67	85.4 ± 16.46	0.398
Operation time (min)	107 ± 75.60	93 ± 20.75	0.730
maintenance time of anesthesia (min)	135 ± 40.57	165 ± 75.26*	0.039

^*^Compared with the young group (*P* < 0.05)

### Total concentration of ropivacaine in plasma

**Fig. 1:**
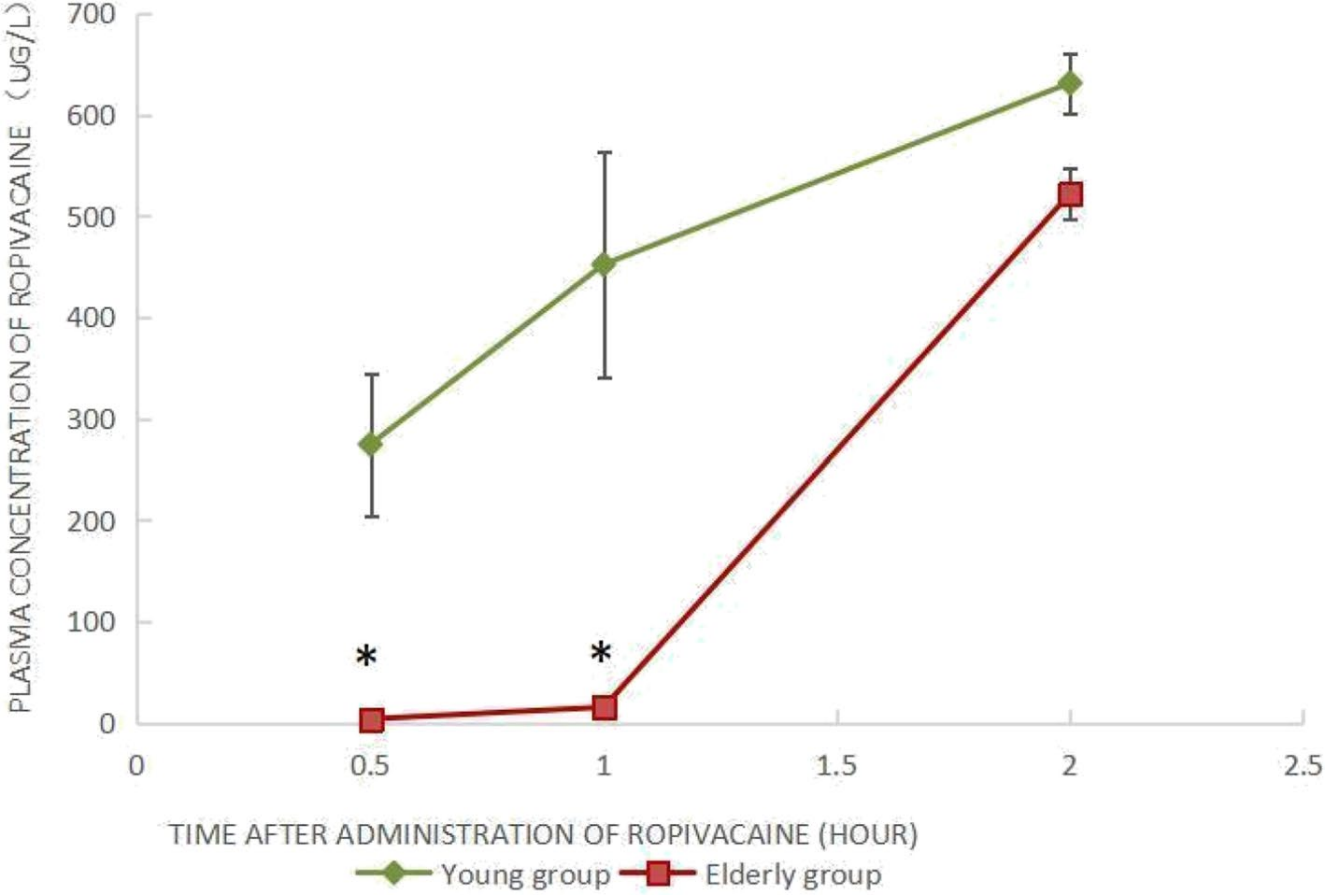
Total concentration of ropivacaine in plasma after ropivacaine administration

Figure 1 shows that at either 0.5 h or 1 h after ropivacaine administration, the plasma concentrations of ropivacaine in the young group were significantly higher than those of the elderly group (Fig. 1, *P < 0.05). However, the difference in plasma concentrations of ropivacaine was insignificant between the two groups at 2 h after spinal anesthesia.

**Fig. 2:**
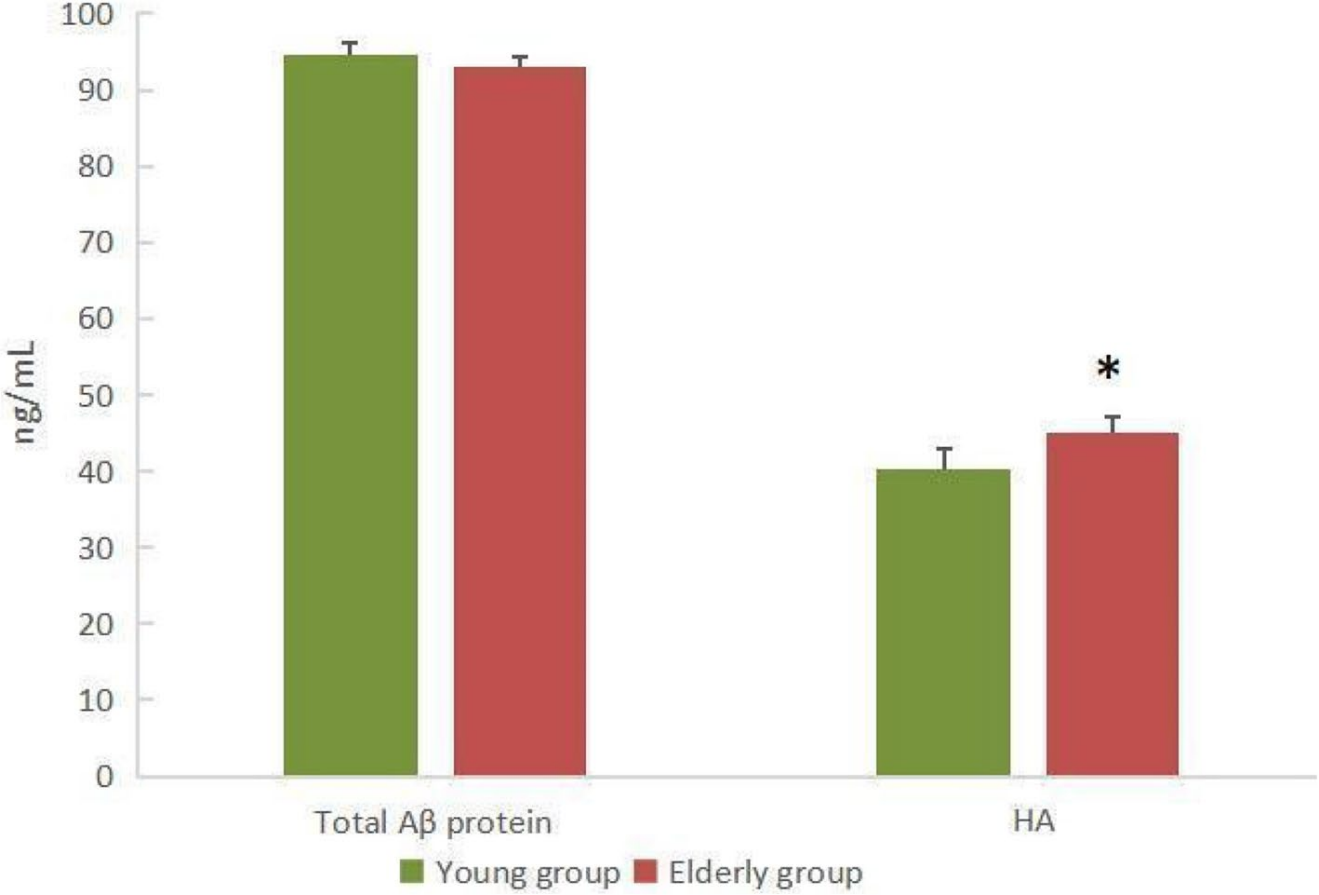
Total Aβ protein and HA levels in CSF in different groups

Figure 2 shows that there was no statistically significant difference in total Aβ protein in CSF between the two groups (93.01 ± 2.49 ng/mL *vs* 94.72 ± 1.42 ng/mL, *P* = 0.261) in all cases. However, HA concentration in the elderly group was noticeably higher than that in the young group(44.99 ± 2.18 ng/mL vs 40.40 ± 1.39 ng/mL, *P* = 0.046).

## Discussion

Our study showed that the concentration of ropivacaine in the peripheral blood of the elderly group increased slowly and the duration of anesthesia maintenance was significantly longer than that of the young patients when there was essentially no difference in renal function and albumin level. This could result from that the metabolism rate of ropivacaine in CSF of an elderly patient is slower than that of a young one. Similarly, Veering et al. (Lanni et al. 2019) have reported that the same dose of bupivacaine is maintained for a longer time during spinal anesthesia in elderly patients, in line with our clinical observations. It is thus suggested that the dosage of ropivacaine for elderly patients should be appropriately reduced during spinal anesthesia and the monitoring time should be appropriately extended.

It is interesting to note that the level of HA in CSF of elderly patients is significantly higher than that of young patients. Due to its high molecular weight (Garnica-Galvez et al. 2021; Fang et al. 2021), the classic metabolic pathway of HA (Wege et al. 2021) is through lymphatic drainage. As a result, the higher level of HA in the elderly group might imply that the CLC (central lymphatic circulation) function of elderly patients is slower than that of young patients. Ma et al. (Ma et al. 2017) proposed that CLC was the major outflow pathway for both large and small molecular tracers in mice. They reported that there was a substantial decline in CSF lymphatic outflow in aged mice compared to young ones, suggesting that the lymphatic system might represent a target for age-associated neurological conditions. As both ropivacaine and HA appear to imitate the degeneration of CLC metabolism in elderly people, promoting CLC and reducing long-term retention of harmful substances in cerebrospinal fluid might be helpful to improve rehabilitation.

This work is clearly a very preliminary study attempting to relate ropivacaine and HA levels with the decline of CLC function in elderly people. Further research work is definitely required to understand the working mechanism, particularly when there is still limited understanding of human CLC.

Genetic studies, biochemical data, and animal models have suggested that Aβ is a critical species in the pathogenesis of cognitive impairment in the elderly [14]. In this work, we did not observe any differences in total Aβ protein level in CSF between the elderly and the young patients, nor did any patients experience postoperative delirium or cognitive impairment. More research is needed on the relationship between spinal anesthesia and postoperative cognitive dysfunction.

In conclusion, our study shows that the metabolism of ropivacaine in cerebrospinal fluid of elderly patients is slower than that of younger patients. It is suggested that the dosage of ropivacaine for elderly patients should be appropriately reduced during spinal anesthesia.

## Data Availability

The datasets used and/or analyzed in the current study are available from the corresponding author upon reasonable request.
